# Design and Optimization of MoS_2_@rGO@NiFeS Nanocomposites for Hybrid Supercapattery Performance and Sensitive Electrochemical Detection

**DOI:** 10.3390/molecules29215195

**Published:** 2024-11-02

**Authors:** Aneeqa Yasmeen, Amir Muhammad Afzal, Areej S. Alqarni, Muhammad Waqas Iqbal, Sohail Mumtaz

**Affiliations:** 1 Department of Physics, Riphah International University, Campus Lahore, Lahore 54000, Pakistan; aneeqakhan123@gmail.com (A.Y.); waqas.iqal@riphah.edu.pk (M.W.I.); 2 Department of Physics, College of Science, Princess Nourah Bint Abdulrahman University, P.O. Box 84428, Riyadh 11671, Saudi Arabia; arsalqarni@pnu.edu.sa; 3 Department of Electrical and Biological Physics, Kwangwoon University, Seoul 01897, Republic of Korea; sohail.ahmed2015@gmail.com

**Keywords:** transition metal dichalcogenide, reduce graphene oxide, two-dimensional, supercapacitors, battery, electrochemical sensor, activated carbon, supercapattery

## Abstract

Metal sulfide-based composites have become increasingly common as materials used for electrodes in supercapacitors because of their excellent conductivity, electrochemical activity, and redox capacity. This study synthesized a composite of NiFeS@MoS_2_@rGO nanostructure using a simple hydrothermal approach. The synthesized nanocomposite consisted of the composite of nickel sulfide and iron sulfide doped with MoS_2_@rGO. A three-electrode cell is employed to investigate the electrochemical properties of the NiFeS@MoS_2_@rGO electrode. The results demonstrated an optimal specific capacitance of 3188 F/g at 1.4 A/g in a 1 M KOH electrolyte. Furthermore, a supercapattery is designed utilizing NiFeS@MoS_2_@rGO//AC as the positive electrode and activated carbon (AC) as the negative electrode materials. The resulting supercapattery is designed at a cell voltage of 1.6 V, achieving a specific capacity value of 189 C/g at 1.4 A/g. It also demonstrated an excellent energy density of 55 Wh/kg with an enhanced power density of 3800 W/kg. Furthermore, the hybrid device demonstrated remarkable stability with a cycling stability of 95% over 30,000 charge–discharge cycles at a current density of 1.4 A/g. The supercapattery, which has excellent energy storage capabilities, is used as a power source for operating different portable electronic devices.

## 1. Introduction

Currently, the ongoing development of electronic devices equipped with advanced technologies and features demands energy storage systems that provide enhanced dependability and improved efficiency [1,2]. Supercapacitors, a form of energy preservation device, have attracted substantial attention because of their ability to charge and discharge quickly, their high energy density, and long life span [3,4]. Supercapacitors, despite recent material advancements that show improved output at a reduced cost, still face challenges because of their poor energy density in comparison to battery materials. This makes the process of commercialization quite challenging. Therefore, the development of electrode materials that possess high specific capacities, significant energy density, and improved rate capability continues to be a challenging and crucial task. Recently, researchers have been intrigued by the development of a novel hybrid energy storage system known as a supercapattery [5]. This system combines the exceptional energy capacity of a secondary battery with the superior power capability and extended charge–discharge process of a supercapacitor [6,7,8,9]. Supercapattery electrode materials generally exhibit more specific energy and power density. This is mostly because of the presence of several oxidation states in the electrode materials, which occur during electrochemical redox processes. The benefit of manufacturing a hybrid device lies in the ability to harness high power from a supercapacitor-type material and merge it with intense energy derived from a substance with battery properties [10]. By combining these two principles, supercapattery devices can result in an increased cell voltage and extended cell lifespan. Transition metal sulfide exhibits distinct characteristics, including high theoretical capacitance, exceptional redox properties, superior electrical conductivity, affordability, and environmental friendliness. These attributes make it highly suitable as an electrode material for supercapacitor applications, instead of traditional transition metal oxides and conductive polymers. ZnS, Cus, NiS, Ni_3_S_2_, MoS_2_, FeS_2_, and CoS are commonly employed in electrodes for supercapacitor devices because of their pseudo-capacitive behavior and superior electrochemical features [11,12,13,14]. The two main types of SCs are electrical double-layer capacitors (EDLCs) and pseudocapacitors. EDLCs accumulate charge through electrostatic separated charges at the boundary where the electrolyte and electrode make contact.

Pseudocapacitors, on the other hand, rely on quick redox responses occurring close to the material on the electrode surfaces [15,16]. Certainly, electrode material is of the greatest significance in the design and manufacturing of a supercapacitor. Hydroxides and transition metal oxides have unique chemical arrangements, physical structures, and excellent pseudocapacitor efficiency and are thoroughly explored as promising electrode materials [17,18,19,20]. To enhance conductivity, many efforts are made to address this challenge [21,22,23]. Furthermore, the combination of multiple metal cations with different valence states enhances the redox couplings, leading to greater capacitance and facilitating electron transfer between atoms, resulting in enhanced electrical potential comparable to that of single metal sulfides. In a recent investigation conducted by Kang et al., created electrodes using mixed metal sulfide [24]. These electrodes exhibited impressive specific capacitances of 1526 and 988 F/g at 2 and 20 A/g, respectively [25,26]. The Ni-Mn sulfide was effectively deposited onto carbon nanofibers, giving rise to free electrodes that are easily modified to specific requirements. These electrodes demonstrate an impressive specific capacity of 652.3 C/g at a 1 A/g current density. These electrodes also demonstrate exceptional current rate qualities; the battery has a capacity retention of 73.6% when the current density is improved by 20 times, and it maintains a cycling reliability of 91.3% after undergoing 5000 cycles, especially when exposed to a substantial current density of 10 A/g [27]. A commonly used technique involves introducing conductive elements, including carbon nanotubes, graphene, and porous carbon, into mixed metal sulfides by a process known as doping [27,28]. Consequently, materials derived from carbon offer advantages. For instance, properties like exceptional conductivity and significant specific surface areas might enhance the flow of charges and boost the quantity of active sites. In addition, the presence of carbon greatly enhances the electrode’s stability by effectively inhibiting the aggregation of molybdenum di sulfide during repeated charge and discharge cycles. However, carbon nanotubes (CNTs) possess notable physical attributes such as a large capacity for transporting electric current, enhanced mechanical properties, and promising electrical conduction capabilities [29]. Hence, the inclusion of synchronized carbon nanotubes (CNTs) and mixed metal sulfide within a (3D) configuration enables the insertion and elimination of ions on the electrode’s surface. A recent study conducted by Fatima et al. that investigated the use of CNTs doping in mixed metal sulfide-based materials, found that CNT@NiCo_2_S_4_ hybrid film has a specific capacitance of 132.2 F/g with a current density of 1 A/g [30].

The cathodes made of iron meet most of these criteria. Therefore, it is recommended to conduct more research on numerous iron-based cathode materials due to their promising economic prospects [31]. The remarkable chemical characteristics of iron-based cathodes have attracted significant attention and prompted extensive research. A comprehensive examination of economically feasible iron-based cathode materials is crucial for a comprehensive evaluation of the recent improvements in such materials for the cathodes of advanced energy storage devices, given their numerous benefits and significant commercial prospects. Recently, Saleem et al. designed a graphene electrode which demonstrates a notable specific capacitance of 1013 F/g at current density of 2 A/g [32]. Based on the above-specified parameters, our team used a hydrothermal process to produce a NiFeS@MoS_2_@rGO nanomaterial. The hydrothermal method is one such adaptable method for making the electrodes for supercapacitors. It is cheap, easy to use, safe for the environment, and allows for a precise crystalline shape with altered diameters and unique pore size distributions. The final NiFeS@MoS_2_@rGO structure has several beneficial properties. Initially, the MoS_2_ significantly increases the surface area, which raises the specific capacitance and increases the ion transportation. Furthermore, the presence of rGO increases the substance’s ductility and, in some cases, minimizes susceptibility to damage to the structure resulting from changes in volume due to expansions and contractions throughout the reaction process.

## 2. Experimental Section

### 2.1. Material

Nickel (II) chloride (NiCl_2_), hydrochloric acid (HCl), activated carbon (AC), iron chloride acetylene black, polyvinylidene difluoride (PVDF), and ethanol were purchased from Sigma-Aldric (Saint Louis, MO, USA). Deionized water was utilized throughout the synthesis procedure. The electrochemical measurement was conducted using a Hg/HgO, platinum wire, and counter electrode, which were acquired from ALS Co., Ltd., Kobayashihigashi, Japan. All the electrochemical measurement was performed using the CS300 electrochemical station. The XRD of model XRD-Rigaku was used. 

### 2.2. Process of Cleaning Ni Foam (NF)

The NF slice (1 × 1 cm^2^) was thoroughly cleaned with a strong HCl solution (0.5 mL) in an ultrasound chamber for 1 min. This process aimed to eliminate the NiO layer that was present on the surface of the Ni foam. Subsequently, it was subjected to further drying in an oven at a temperature of 80 °C for one night.

### 2.3. Synthesis Process

#### 2.3.1. Preparation of Nickel–Iron Sulfide

**Figure 1:**
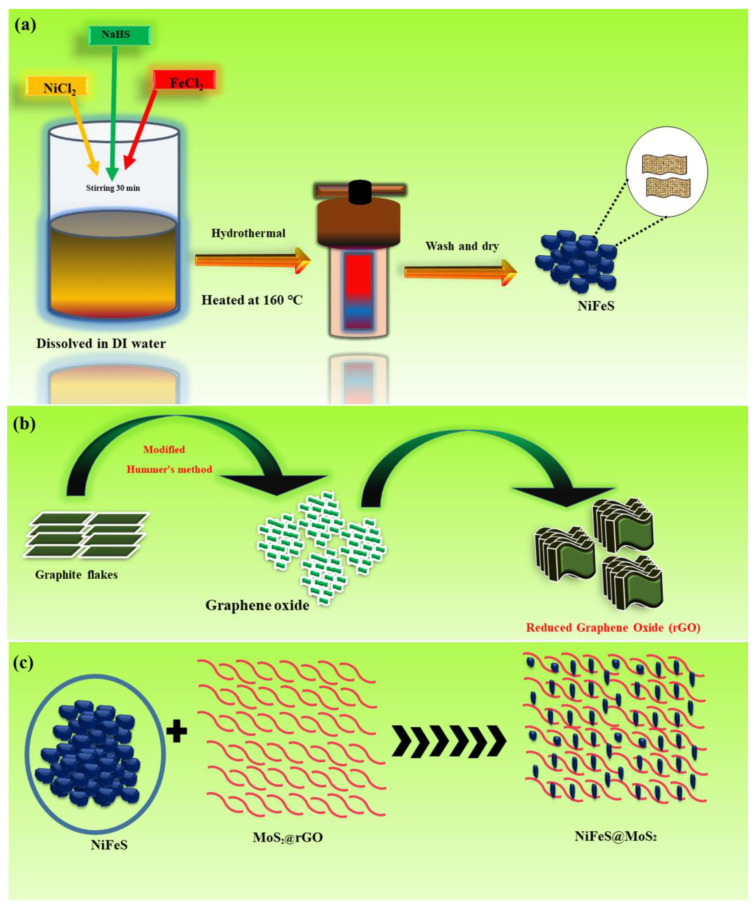
(**a**) Represents the procedure of syntheses of NiFeS; (**b**) procedure of the fabrication of the rGO; (**c**) process of the NiFeS@MoS_2_@rGO.

The nickel sulfide nanomaterial was manufactured utilizing the hydrothermal technique. First, the solution of nickel (II) chloride, with a concentration of 0.8 M was combined with 50 mL of deionized water. The solution was positioned upon the magnetic stirrer and mixed for 30 min at room temperature. The sodium hydrosulfide solution at a concentration of 0.8 M was dissolved with 50 mL of deionized water and mixed for 30 min at room temperature. Similarly, a 2nd solution containing concentrations (0.8 M) of iron chloride and sodium hydrosulfide was prepared by mixing them in 50 mL of deionized water and mixed for 30 min on a magnetic stirrer. Subsequently, both solutions were mixed drop wise and stirred for 30 min to make a 3rd solution which was place into the oven at a temperature of 160 °C for 8 h. Subsequently, the solution underwent multiple washes in a centrifuge utilizing ethanol and deionized water (DI) to minimize the presence of contaminants for 10 min, and dried at room temperature for one day. The procedure of nickel sulfide and iron sulfide is illustrated in Figure 1a.

#### 2.3.2. Synthesis of rGO

The fabrication of graphene oxide (GO) occurred using an improved version of Hummer’s process.

In a 1 L glass beaker, 350 mL of H_2_SO_4_, 50 mL of H_3_PO_4_, 4 g of graphite flakes, and 19 g of (KMnO_4_) were thoroughly mixed. Through continuous agitation over 48 h at ambient temperature, the graphite underwent complete oxidation. The mixture transformed from a dark green hue to a dark brown shade. The process of oxidation was interrupted by employing ice-carrying H_2_O_2_ to regulate the mixture’s temperature. Graphite oxide was purified by centrifugation, which extracts and eliminates the liquid part (supernatant) with each revolution [33,34]. The graphite oxide underwent exfoliation through washing with deionized water, resulting in the formation of a concentrated graphite solution and ultimately yielding a gel of graphene oxide (GO). The procedure is shown in Figure 1b.

#### 2.3.3. Preparation of Nickel–Iron Sulfide@MoS_2_@rGO Composite

The nickel sulfide and iron sulfide were doped with MoS_2_@rGO physical blending at 50/50 weight percentages. Figure 1c illustrates the combination of nanomaterial and the fabrication of composite material.

#### 2.3.4. Synthesis of Electrode Materials and Fabrication of Electrodes

To conduct the electrochemical tests, the electrodes are made by applying a layer of the active materials onto chemically treated nickel foam. The active material slurry was prepared by combining the NiFeS@MoS_2_@rGO nanocomposite (80%), AC (10%), and PVDF (10%) in NMP. The mixture is then agitated for 8 h on a magnetic stirrer at room temperature to ensure uniformity. The slurry is applied on the NF using drop coating, and then it is placed at a temperature (80 °C) for 9 h to dry. The entire loading of the active substance on all produced electrodes was approximately 5.00 ± 0.05 mg.

### 2.4. Electrochemical Measurements

The suitability of NiFeS@MoS_2_@rGO for the supercapattery application is assessed by utilizing a three-electrode cell in a 1 M KOH solution at room temperature. The active material is applied to a 1 × 1 cm^2^ nickel foam surface. It functioned as the working electrode. The electrode is employed as the reference Hg/HgO. A platinum wire serves as the counter electrode.

## 3. Results and Discussion

**Figure 2:**
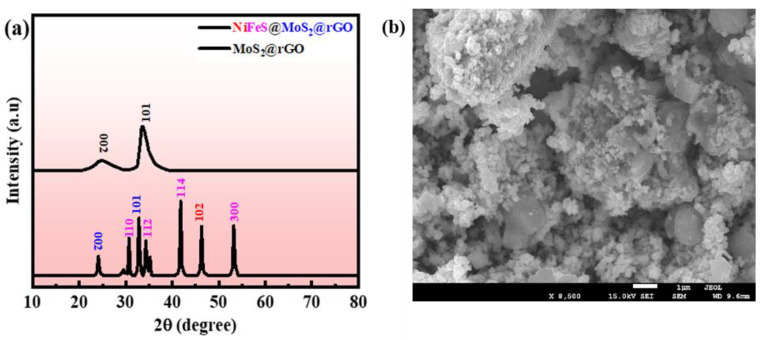
(**a**) XRD of NiFeS@MoS_2_@rGO; (**b**) SEM image of composite NiFeS@MoS_2_@rGO.

The NiFeS@MoS_2_@rGO is analyzed using an XRD spectrum, as revealed in Figure 2a.

The image shows a strong diffraction pattern for NiS, demonstrating a bigger crystallite nature in the sample. The diffraction peaks for NiS are identical (JCPDS card no. 89-7141). The (102) plane of NiS crystal corresponds to diffraction peaks of 46.5°. The FeS (JCPDS No. 65-3356) planes are allocated to the peaks at 28.6°, 29.9°, 33.7°, 43.2°, and 53.1°, respectively, as planes (103), (110), (112), (114), and (300). The rGO diffraction peaks are identical at 2θ = 26.8°, to plane (002). MoS_2_ is found to have two main peaks at 33.3°, which are correspondingly indexed to (1 0 1) planes. It aligns with JCPDS 37-1492 [35,36,37]. The Scherer equation is used to estimate the size of nanostructure crystals:

(1) $$D=\frac{K\lambda }{\beta CoS\theta }$$

Here, *β*, *K*, *λ*, and *θ* signify the constant term. The materials synthesized have crystal diameters of 62 and 81 nanometers, correspondingly. Figure 2b illustrates the (SEM) image of NiFeS@MoS_2_@rGO. Raman spectra for NiFeS@MoS_2_@rGO is shown in Figure S1.

**Figure 3:**
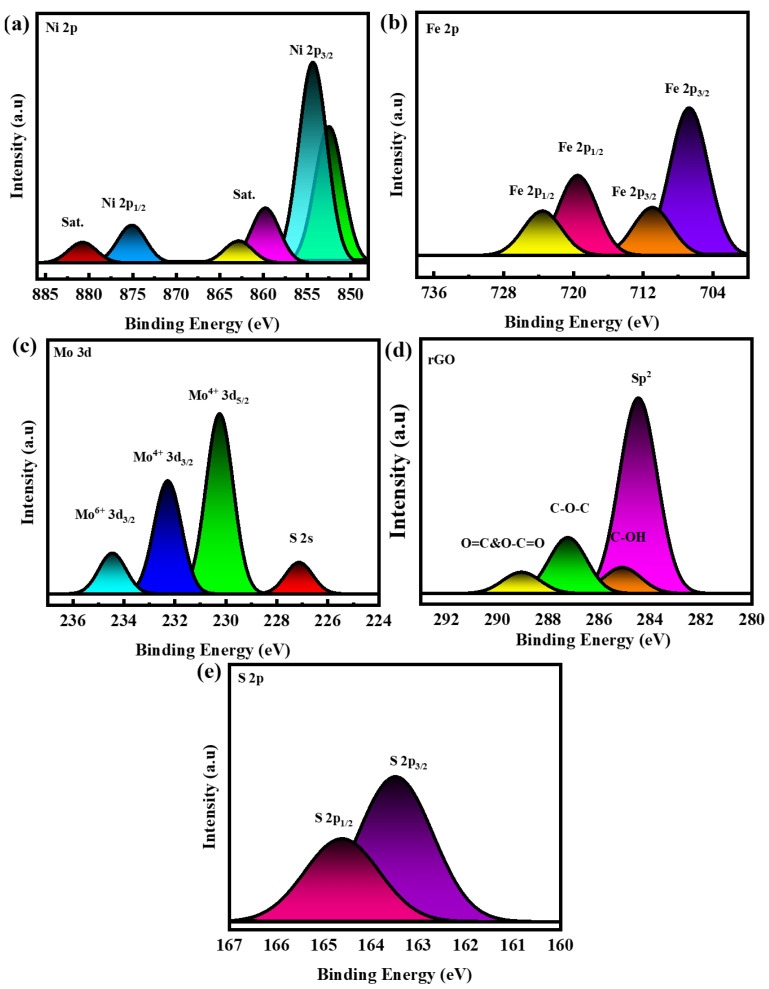
(**a**) XPS of Ni 2p; (**b**) Fe 2p XPS; (**c**) MoS_2_ XPS; (**d**) XPS of rGO; (**e**) S 2p XPS.

The results indicate that the addition of MoS_2_@rGO to the reference samples enhances the material’s porosity. Figure 3a shows the XPS survey spectrum of nickel sulfide.

The nickel sulfide exhibits peaks at 855.9 eV for Ni 2p_3/2_ and 875.4 eV for Ni 2p_1/2_ in its Ni 2p spectra, which correspond to Ni_3_S_2_ and two shake-up satellites. The Fe 2p binding energy peaks at 704.1 eV and 717.1 eV correspond to Fe 2p_3/2_ and Fe2p_1/2_ binding energy, respectively. The peaks at 709.6 eV and 725.1 eV correspond to (Fe-S)2p_3/2_ and (Fe-S)2p_1/2_ binding energies demonstrated in Figure 3b. The Mo 3d binding energy maxima at 230.5 and 232.1 eV relate to 2H MoS_2_ with Mo^4+^ 3d_5/2_ and Mo^6+^ 3d_3/2_ oxidation states. The peaks at 234.3 eV correspond to Mo^6+^ 3d_3/2_ shown in Figure 3c [36]. Figure 3d shows spectra of GO showed a high C/O ratio of 1.33. Spectrum peaks at 284.5, 285.4, 286.6, 287.8, and 288.8 eV correspond to C–C, C–C, C–O, C–O, and O–C=O groups [38]. Figure 3e shows that the S2p spectrum is divided into two peaks. The two largest peaks at 163.9 and 165.0 eV correspond to the S 2p_3/2_ and S 2p_1/2_ spectra [39]. The full XPS survey spectrum of NiFeS@MoS_2_@rGO is shown in the Figure S2.

### 3.1. Brunauer–Emmett–Teller (BET)

**Figure 4:**
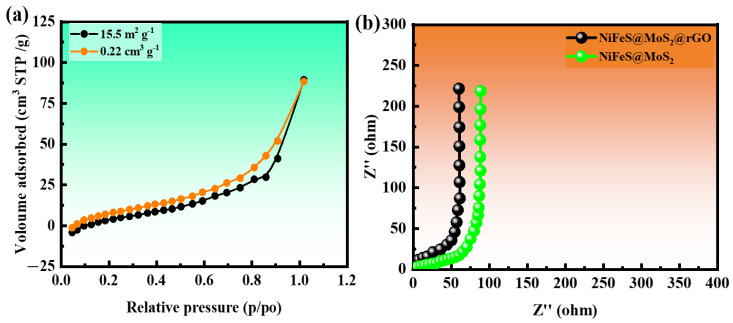
(**a**) BET analysis for NiFeS@MoS_2_@rGO; (**b**) EIS measurements for NiFeS@MoS_2_ and NiFeS@MoS_2_@rGO.

The Brunauer–Emmett–Teller (BET) process is employed to examine a specific surface area (SSA) by continually collecting nitrogen gas onto a solid specimen. The restricted adsorption of the molecule on the gas surface is due to the weak van der Waals interactions that create a monolayer of gas. The surface area, pore size, and pore volume of NiFeS@MoS_2_@rGO are estimated using BET calculations. Figure 4a demonstrates that the shape of the BET isotherm curves closely resembled that of an IV-type graph.

The specific surface area (SSA) and pore volume and pore size of NiFeS@MoS_2_@rGO are determined to be 15.5 m^2^ g^−1^ 0.022 cm^3^ g^−1^ and 2.9 nm to 5 nm, respectively. The surface area, pore volume, and pore size for NiS are 9.0 m^2^ g^−1^, 0.15 cm^3^ g^−1^, and 1.5–3 nm, and for FeS 10.2 m^2^ g^−1^, 0.16 cm^3^ g^−1^ and 2.1–4 nm shown in Figure S3. The remarkable electrochemical performance of NiFeS@MoS_2_@rGO is attributed to its substantial surface area. The electrochemical storage capacity is enhanced by the mesoporous structure of NiFeS@MoS_2_@rGO. The findings indicate that an increased surface area promoted the accumulation of charges, while a greater volume supported the rapid transportation of ions.

### 3.2. Electrochemical Impedance Spectroscopy (EIS)

Electrochemical impedance spectroscopy (EIS) studies are conducted in the frequency interval of 0.1–100 kHz to assess the conductance of NiFeS@MoS_2_@rGO to obtain information about the chemical reaction occurring at the interface. Figure 4b shows that the Nyquist charts for NiS, FeS, NiFeS@MoS_2_, and NiFeS@MoS_2_@rGO do not have the typical semicircular shape. This suggests that the unequal electrochemical activity is caused by the control of the electric field and the texture of the electrode surface. Equivalent series resistance (ESR) is the resistance that occurs at the point when the electrode and electrolyte come into contact. The Nyquist graphs are utilized to compute the equivalent series resistance (ESR) at the intersection of higher frequencies on the x-axis. ESR is also utilized to denote the aggregate resistances, encompassing the resistance related to current collection, connection, and electrodes. The recorded ESR (electron spin resonance) values NiS, FeS, NiFeS@MoS_2,_ and NiFeS@MoS_2_@rGO are 1.25, 1.2, 1.08, and 0.80 Ω shown in Figure S4, respectively.

### 3.3. Electrochemical Studies

#### 3.3.1. Cyclic Voltammetry Studies

**Figure 5:**
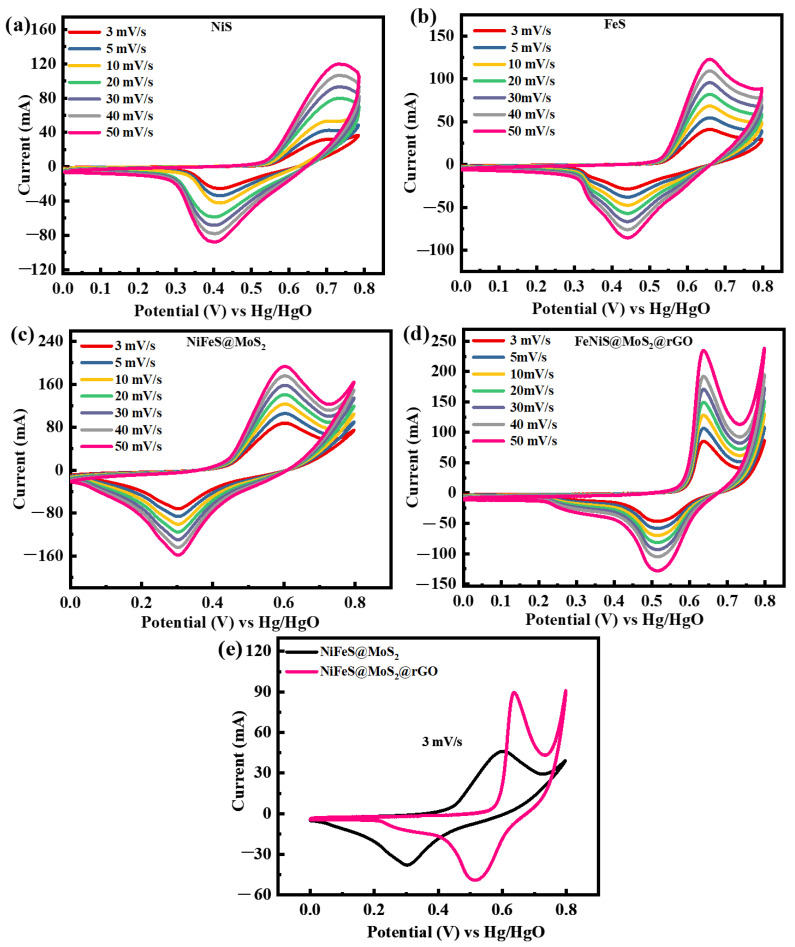
(**a**) CV measurements of NiS; (**b**) analysis of FeS at the scan rate of 3–50 mV/s; (**c**) CV of NiFeS@MoS_2_; (**d**) CV profile for the ternary composite NiFeS@MoS_2_@rGO at varying scan rates (3 mV/s to 50 mV/s); (**e**) comparison of both electrodes NiFeS@MoS_2_ and NiFeS@MoS_2_@rGO at 3 mV/s.

Cyclic voltammetry (CV) is a technique used to assess the electrochemical characteristics of nanomaterials. The morphology of the cyclic voltammetry (CV) curves is employed to elucidate the properties of the material. Once the negative polarity is increased, they ultimately hit a threshold where the liquid contains far fewer electrons than the electrode. Consequently, electrons undergo a reduction current, moving from the electrode to the solution, namely from a higher to a lower potential. The reduction process is the primary cause of the faradaic current in this case. When the positive potential is increased, the electrons are subsequently shifted from the mixture to the electrode. The movement of electrons generates a current recognized as the oxidation current [40]. CV measurements are utilized to investigate the properties of the fabricated NiS, FeS, NiFeS@MoS_2_, and NiFeS@MoS_2_@rGO using an operating potential (OP) of 0.8 V. The acquired outcomes are demonstrated in Figure 5a–d. The CV curve for the perfect EDLCs is a rectangular shape, whereas, for PCs, the CV curves displayed tiny faradic peaks interspersed within the rectangular pattern. The electrode substance exhibits enhanced catalytic activity based on the proximity of the redox reaction peaks during the charge storage process. These considerations elucidate the genesis of the faradic responses. If the redox peaks align perfectly, the process is reversible. Assuming the presence of a small discrepancy, the mechanism is semi-reversible. And if the peaks are distinct, the process is irreversible. The presence of both faradaic and nonrectangular CV forms in these materials suggests that they have undergone battery grading.

The dispersion of electrolytes is additionally associated with redox peaks, suggesting that NiS, FeS, NiFeS@MoS_2_, and NiFeS@MoS_2_@rGO displayed battery-like properties. The NiS material is manually combined with MoS_2_@rGO to enhance its optimization. The operating potential of 0.8 V is sustained, and cyclic voltammetry (CV) calculations are performed at altered scan rates ranging from 3 to 50 mV/s. The electrochemical activity of binary NiFeS is enhanced as an outcome of the synergistic influence of MoS_2_@rGO and NiFeS [41]. The synergistic influence of MoS_2_@rGO and NiFeS leads to a rise in the number of active sites, surface area, and pores, resulting in improved electrochemical activity. The results demonstrate that the combination of MoS_2_@rGO with NiFeS enhances the materials’ surface area and conductivity, both of which are crucial factors for electrochemical characteristics. The surface area enclosed by the CV curve expands as the scan rate increases [42]. Figure 5e presents an analysis of CV (cyclic voltammetry) for NiFeS@MoS_2_ and NiFeS@MoS_2_@rGO at a scan rate of 3 mV/s. It provides evidence supporting the claim that the material NiFeS@MoS_2_@rGO exhibits strong redox peaks. The relationship between the amount of charge stored on electrodes and the area under cyclic voltammetry (CV) curves is widely recognized. NiFeS@MoS_2_@rGO shows a wider surface area when compared with NiFeS@MoS_2_. The significant distinction emphasizes the outstanding electroactive properties of NiFeS@MoS_2_@rGO, indicating its improved ability to store charges and its potential for many electrochemical applications.

#### 3.3.2. Galvanostatic Charge–Discharge Studies

**Figure 6:**
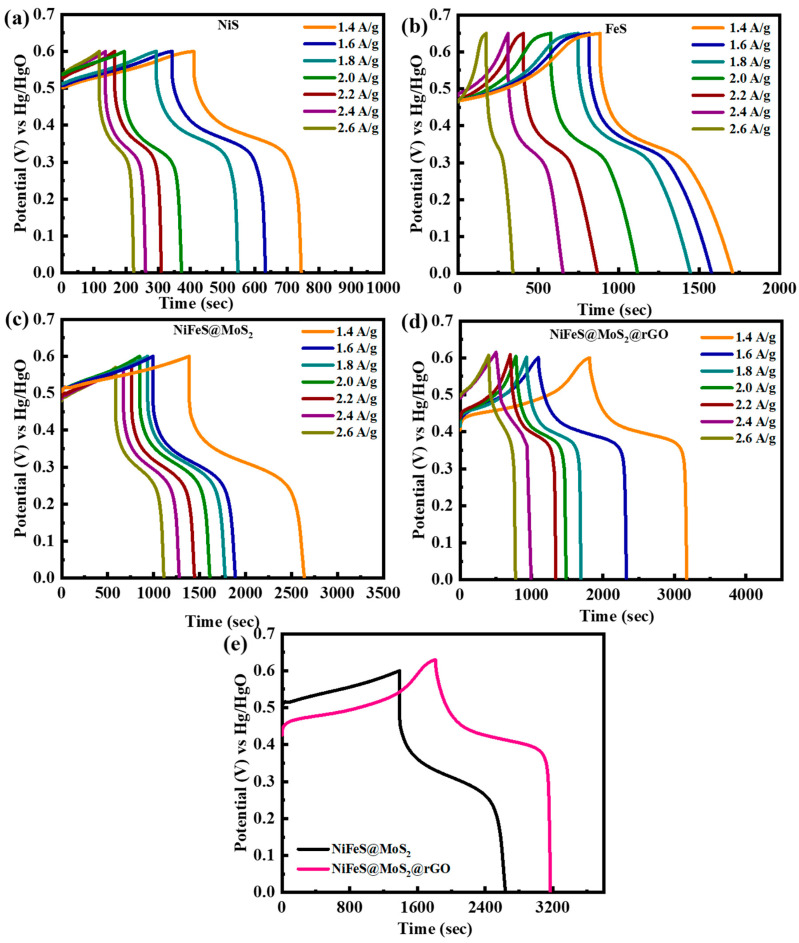
(**a**) GCD illustration for NiS; (**b**) GCD measurements for FeS; (**c**) GCD graphs for the composite NiFeS@MoS_2_ at a current density alternating from 1.4 to 2.6 A/g; (**d**) GCD pattern for NiFeS@MoS_2_@rGO at current densities ranging from 1.4 to 2.6 A/g; (**e**) NiFeS@MoS_2_ and NiFeS@MoS_2_@rGO electrodes comparison at 1.4 A/g.

To determine the stability of the electrodes NiFeS nanocomposite doped with MoS_2_@rGO nanoparticles, galvanostatic charge–discharge experiments are executed. The GCD pattern in the range of 1.4 to 2.6 A/g at a potential of 1.6 V is revealed in Figure 6a–d at numerous current densities. The quasi-symmetric discharge curves attest to the pseudo-capacitive nature of the electrodes, which is also supported by the CV pattern. Redox reactions happen at the contact point between the electrode and electrolyte.

When compared to other electrodes, the NiFeS@MoS_2_@rGO nanocomposite exhibits an extended discharge period. In the instance of the MoS_2_@rGO nanocomposite, NiFeS is cultivated on a graphene matrix that is evenly dispersed. This cultivation reduces clumping and enhances the number of electroactive sites. Due to its conductive plate structure, graphene boosts the electrochemical effectiveness of the material. The boosted performance of the NiFeS@MoS_2_@rGO is attributed to the additional decorating of MoS_2_@rGO nanoparticles, which contributes to the electrochemical redox reaction and improves its efficiency. The extended duration of discharge for NiFeS@MoS_2_@rGO signifies the superior capacity of MoS_2_@rGO. The simultaneous GCD curves of NiFeS@MoS_2_@rGO exhibited the optimal oxidation/reduction properties and substantial capacity due to the extended discharge duration. The outstanding conductivity and comparatively minimal resistance of all materials also resulted in minimal IR loss, even at very extraordinary densities [43]. The discovery suggested that the substance exhibits prominent interconnected redox peaks when undergoing charge–discharge processes, indicating the occurrence of faradaic reactions. Figure 6e presents a comparison of NiFeS@MoS_2_ and NiFeS@MoS_2_@rGO. It demonstrates that NiFeS@MoS_2_@rGO exhibited a longer discharge time compared to the other materials. The extended discharge duration of NiFeS@MoS_2_ and NiFeS@MoS_2_@rGO signifies their superior capacity compared to other materials. The extended discharge lifespan of NiFeS@MoS_2_ and NiFeS@MoS_2_@rGO compared to other produced materials is attributed to a significant surface area exposed to OH^−^ ions. The specific capacity and capacitance are determined by examining the CV and GCD curves, and the relationship is calculated using Equations (2) and (3).

(2) $$Q_{s}=\frac{1}{mv}\int_{vi}^{vf}I\times VdV$$

*I* symbolizes the current in the provided equation, which remains constant through the charge–discharge process. *Q_s_* represents the specific capacity, measured in C/g. The variable *m* denotes the mass of the active material, namely the mass of the synthesized nanomaterials put on the electrode.

(3) $$Q_{s}=\frac{I\times t}{m}$$

*I* describes the presence of current and *t* represents the duration of the discharge. The energy and power density are determined through the utilization of Equations (4) and (5).

(4) $$E_{d}=\frac{C_{g}(\Delta V)}{2\times 3.6}$$

(5) $$P_{b}=\frac{E_{d}\times 3600}{t}$$

**Figure 7:**
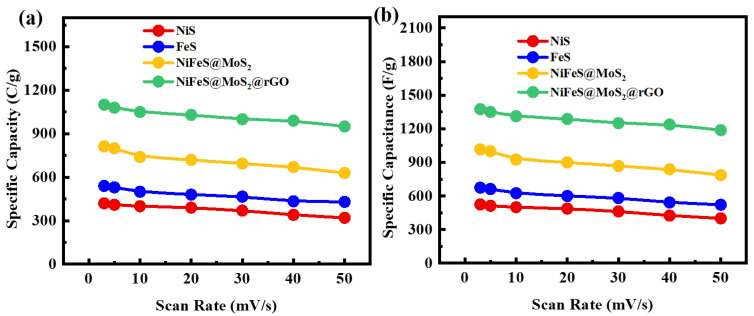
(**a**) Specific capacity of NiS, FeS, NiFeS@MoS_2_, and NiFeS@MoS_2_@rGO; (**b**) specific capacitance at 3 to 50 mV/s scan rate for all samples.

**Figure 8:**
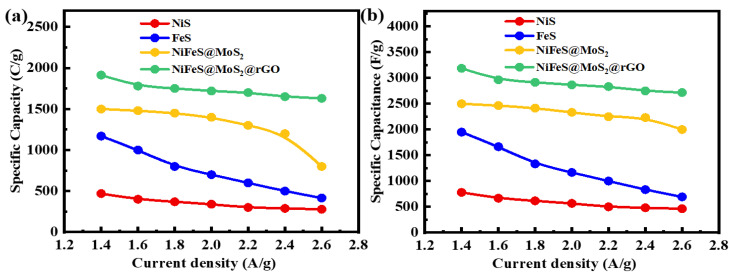
(**a**) Examination of specific capacity at different current densities, alternating from 1.4 A/g to 2.6 A/g; (**b**) specific capacitance of the GCD graph for all samples across a current density that varies from 1.4 A/g to 2.6 A/g.

*C_g_* refers to the specific capacity acquired from a GCD pattern. The variable *t* in Equation (5) denotes the duration required for the discharge. The above equation demonstrates that a sample with a larger area under the CV curve exhibits a greater charge storage capacity at any given scan rate. Figure 7a,b displays the specific capacities and specific capacitance of all the samples, which are determined through the calculation of the CV profile at the relevant scan rate. The specific capacities for NiS, FeS, NiFeS@MoS_2_, and NiFeS@MoS_2_@rGO are 420 C/g, 540 C/g, 813 C/g, and 1100 C/g, respectively. The decline in specific capacity when the scan rate is raised is due to the reduced chance for electrolyte ions to be involved with the active electrode material. The specific capacitance values obtained from the CV profiles for NiS, FeS, NiFeS@MoS_2_, and NiFeS@MoS_2_@rGO are 525 F/g, 665 F/g, 1016 F/g, and 1375 F/g. Figure 8a,b demonstrates the *Q_s_* obtained from GCD measurements for NiS, FeS, NiFeS@MoS_2_, and NiFeS@MoS_2_@rGO which are (469 C/g), (1170 C/g), (1500 C/g), and (1913 C/g). The specific capacitance values acquired from the GCD profile for NiS, FeS, NiFeS@MoS_2_, and NiFeS@MoS_2_@rGO are 781 F/g, 1950 F/g, 2500 F/g, and 3188 F/g. The electrochemical measurements for various nanostructures and their hybrid analogs are shown in Table 1.

### 3.4. Electrochemical Performance of NiFeS@MoS_2_@rGO//AC Supercapattery

**Figure 9:**
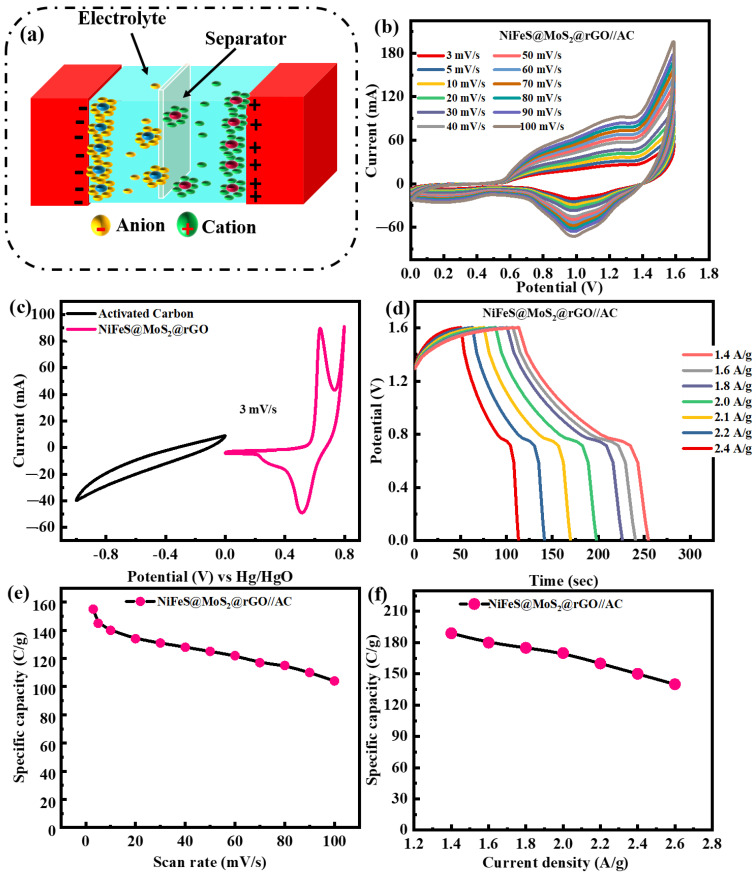
(**a**) Illustrates a two-electrode configuration (NiFeS@MoS_2_@rGO//AC); (**b**) the CV pattern at scan rates alternates from 3 to 100 mV/s; (**c**) CV analysis of AC and NiFeS@MoS_2_@rGO for three-electrode configurations; (**d**) GCD curves for real devices; (**e**) specific capacity measurements for all samples; (**f**) graph of specific capacity for all samples at a current density alternating from 1.4 to 2.6 A/g.

A supercapattery is a device that merges the unique characteristics of a capacitor and a battery. In the area of a supercapattery, increasing the range of the operational voltage is crucial for increasing the energy density, which serves as the primary factor. A supercapattery is designed to utilize a ternary nanocomposite, NiFeS@MoS_2_@rGO at 1.4 A/g, as the positive electrode and AC as the positive electrode (NiFeS@MoS_2_@rGO//AC), as shown in Figure 9a. During the electrochemical investigations, the initial step involved conducting separate CV for the ternary nanocomposite, NiFeS@MoS_2_@rGO. Hence, the device’s maximum stable operating potential is maintained at 0 to 1.6 V. Figure 9b displays the CV of NiFeS@MoS_2_@rGO//AC at altered scanning rates (3 to 100 mV/s) within a potential window of 0 to 1.6 V. It is evident that there are no peaks observed in the CV for the supercapattery. This indicates that the ACs play a role in accumulating energy at the boundary between the electrode and electrolyte within this specific range of potential, including the process of adsorbing and inserting electrolyte ions.

Therefore, as the voltage exceeds, distinct peaks become visible in the CV indicating the initiation of redox reactions that play a role in the energy storage device.

The supercapattery demonstrates the unique characteristics that combine a capacitor and a battery while accumulating charge. The discussion above clarifies that the performance of the assembled device depends on two mechanisms. Energy storage is primarily due to capacitive or non-faradic behavior. The form, consistency, and magnification CV conducted at numerous scanning rates (3 to 100 mV/s) exhibited superior stability. Figure 9c demonstrates the CV analysis of NiFeS@MoS_2_@rGO and activated carbon at 3 mV/s for three-electrode configurations.

Figure 9d illustrates the charge–discharge forms of the fabricated NiFeS@MoS_2_@rGO//AC device at numerous current densities, varying from 1.4 to 2.6 A/g, across a potential range of 1.6 V. The charge–discharge graph exhibits symmetrical patterns, which indicate the presence of capacitive behavior along with reversible redox processes. Figure 9e shows the specific capacity by CV measurements that is 155 C/g. The specific capacity value of NiFeS@MoS_2_@rGO//AC is 189 C/g at a current density of 1.4 A/g and decreases to 140 C/g when the current density is increased to 2.6 A/g shown in Figure 9f. The two-electrode assembly for NiS//AC, FeS//AC and NiFeS@MoS_2_//AC for CV and GCD analysis are shown in the Figure S5. The specific capacity by CV and GCD measurement are shown in Figure S6. The values for specific capacity obtained from the CV measurement for NiS//AC, FeS//AC and NiFeS@MoS_2_//AC are 65 C/g, 72 C/g and 100 C/g. And for GCD measurement specific capacity, the values are 70C/g, 80 C/g, and 110 C/g shown in Table 2.

The CV results are subjected to a model proposed by Dunn et al., [47] which establishes a correlation between scan rate and current:

(6) $$i(v)=k_{1}v+k_{2}v^{0.5}$$

*k*_1_ and *k*_2_ are constants, characteristic that the device’s capacity is obtained by capacitive and diffusive processes at a certain scan rate. In Equation (6), *k*_1_*v* symbolizes the impact of current density using the capacitive-controlled approach, whereas *k*_2_*v*^0.5^ represents the influence of current density employing the diffusion-controlled method.

(7) $$\frac{i(v)}{v^{0.5}}=k_{1}v^{0.5}+k_{1}$$

**Figure 10:**
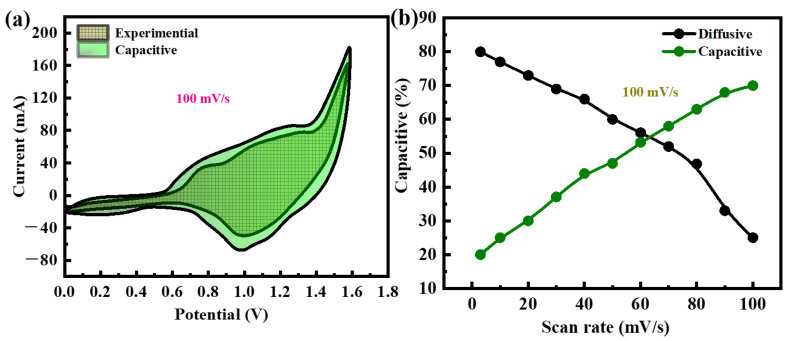
(**a**) The experimental CV of the device at 100 mV/s; (**b**) the capacitive and diffusive contributions are depicted in a bar graph at 100 mV/s.

**Figure 11:**
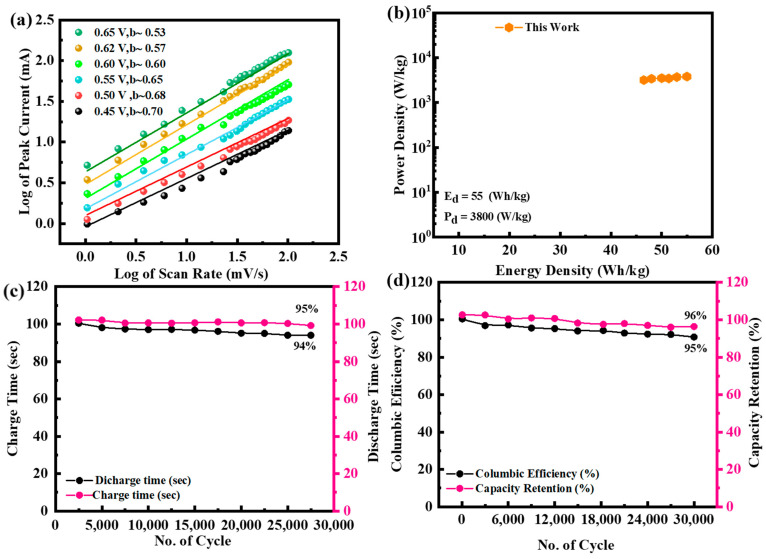
(**a**) Displays the b-value graph for NiFeS@MoS_2_@rGO; (**b**) measurements of energy and power density of NiFeS@MoS_2_@rGO; (**c**) observations of the charging and discharging process of NiFeS@MoS_2_@rGO completed during 30,000 cycles; (**d**) this analysis of columbic efficiency and capacity retention of the NiFeS@MoS_2_@rGO sample.

Graphing the constants *k*_1_ and *k*_2_ as a function of *v*^0.5^ allows for their determination. The current impact formed by diffusive and capacitive regulated processes is shown graphically in Figure 10a,b at 100 mV/s. At a scan rate of 100 mV/s, the capacitive influence is 70% and the diffusive impact is 30%. The b-value fitting at various potential values is displayed in Figure 11a. For the real device, the b-values range from 0.5 to 0.65. When the b-value falls somewhere between 0 and 0.5, it shows a battery-type device; when the b-value falls somewhere between 0.8 and 1.0, it indicates capacitive behavior. A hybrid device (the supercapattery) is demonstrated by the intermediate b-value of 0.5 to 0.8. The device’s computed b-values support the supercapattery claim even more. The energy density and power density of the device are measured at 55 Wh/kg and 3800 W/kg shown in Figure 11b. The device is measured at 30,000 charging and discharging cycles to ensure lifelong stability. During the investigation, the current density of the devices remained constant. The charge–discharge period following 30,000 cycles is shown in Figure 11c.

Figure 11d illustrates that the built supercapattery electrode maintains 95% of its specific capacity after undergoing 30,000 cycles. The outstanding stability of the synthesized supercapattery, attained through the utilization of a modified electrode including a ternary nanocomposite, ensures its logical implementation.

## 4. Conclusions

The synthesis of NiFeS@MoS_2_@rGO nanocomposite for electrochemical storage application is achieved using a straightforward one-step hydrothermal procedure. The effective synthesis and characterizations are confirmed using XRD, SEM, and XPS techniques. The NiFeS is cultivated on the MoS_2_@rGO framework. The electrochemical performance of the material is significantly enhanced when it is doped with MoS_2_ and rGO, as demonstrated through tests using CV, GCD, and EIS. The built supercapattery (NiFeS@MoS_2_@rGO//AC) exhibited a significant energy density of 55 Wh/kg at a power density of 3800 W/kg, attributed to the synergistic effect of MoS_2_ and rGO combined with AC. Furthermore, the study demonstrated a significant specific capacity of 189 C/g at 1.4 A/g. It also has outstanding reversibility, with a cycling efficiency of approximately 95% after 30,000 cycles. NiFeS@MoS_2_@ rGO nanocomposite possesses the capability to work as an alternative electrode material in supercapattery applications and facilitates the advancement of high-performance electrochemical storage devices.

## 5. Electrochemical Characterization of Sensors

**Figure 12:**
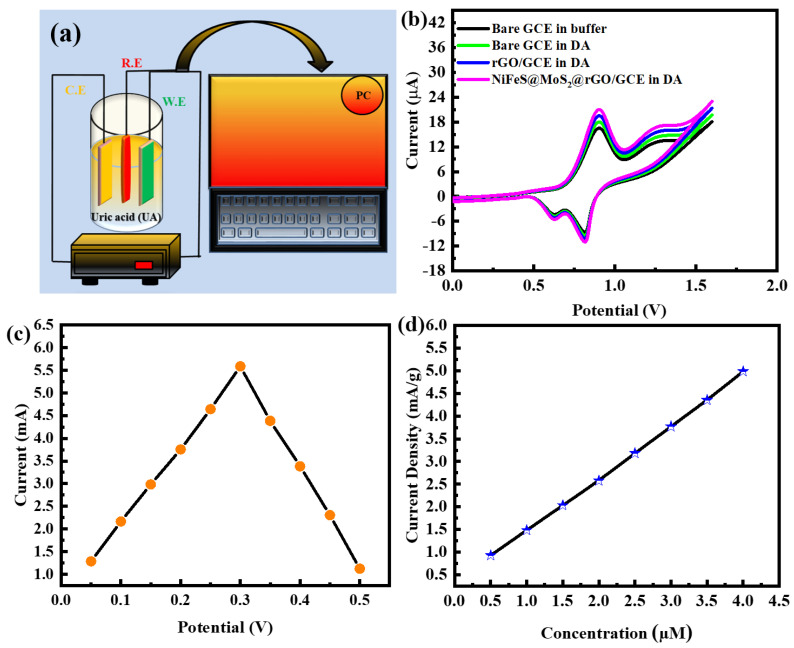
(**a**) Shows a representation of uric acid; (**b**) bare and glassy carbon electrode in a solution of dopamine (DA) CV; (**c**) potential response; (**d**) the graph displaying the results of the measurement for DA.

Figure 12a illustrates the presentation of uric acid. The three electrodes’ cyclic voltammetry profiles are illustrated in Figure 12b. A glassy carbon electrode [48] deep in a buffer solution and the presence of DA caused distinct redox peaks to emerge on all three electrodes. The NiFeS@MoS_2_@rGO@GCE improved sensing capability is noticeable. The sensing effectiveness of the rGO@MoS_2_@NiFeS@GCE sensor is better in a DA solution than in that of the GCE without wrapping and the rGO/GCE sensors, according to the results of the experiment. A shorter time between the waveform’s peak and fall and a better response to electrical current are indications of this. The improved capability to identify dopamine (DA) utilizing a glassy carbon electrode [48] modified with rGO@MoS_2_@NiFeS@GCE can mainly be ascribed to multiple variables. The factors that contribute to the properties of the NiFeS@MoS_2_@rGO@GCE characteristics that set them apart from other materials are their high electrical conductivity, low resistance to charge flow, and the presence of functional groups that act negatively, including hydroxyl and fluorine. The relationship between the operating voltage variation and the amperometric response of dopamine (DA) is demonstrated in Figure 12c. The repeatability, stability, and reproducibility of the sample are shown in the Figure S7. The DA revealed a matching rise as the potential rose progressively from 0.150 to 0.250 V. Nevertheless, further increases in the potential led to a reduced current reaction. As a result, the potential measurement of 0.245 V, the highest favorable current reaction, is selected for an assessment of DA. Figure 12d illustrates NiFeS@MoS_2_@rGO sensor-based correlation of current reaction with dopamine (DA) concentrations ranging from 0.015 to 10 mM. A correlation value of 0.998 has been observed for this connection. The estimation of the limit of detection (LOD) depends on the results that are achieved.

## Figures and Tables

**Table 1:** Electrochemical characteristics of different and hybrid nanostructures.

Sr.No	Samples	Methods	Electrolyte	Specific Capacitance	Cycle Life	Ref.
1	Nanoporous net-like Ni_3_S_2_ thin film	Electrodeposition	1 moL.^−1^ KOH	7.25 F·cm^−2^ at 5 mA·cm^−2^	77%/5000 0.05 mA·cm^−2^	[44]
2	V_2_O_2_/Ni_3_S_2_ nanoflake	Hydrothermal	2 moL.^−1^ KOH	4.2 F·cm^−2^ at 5 mA·cm^−2^	85%/2500 10 mA·cm^−2^	[45]
3	Hierarchical Co_3_@NiS_2_ core/shell nanowire arrays	Hydrothermal	3 moL.^−1^ KOH	1710 F·g^−1^ at 1 A·g^−1^	85%/1000 4 A·g^−1^	[46]

**Table 2:** Two-electrode assembly measurements for NiS//AC, FeS//AC, and NiFeS@MoS_2_//AC.

Sr.No	Sample	Specific Capacity (CV)	Specific Capacity (GCD)
1	NiS	65	70
2	FeS	72	80
3	NiFeS@MoS_2_	100	110

## Data Availability

The original contributions presented in the study are included in the article/Supplementary Material, further inquiries can be directed to the corresponding author.

## Supplementary Materials

The following supporting information can be downloaded at: https://www.mdpi.com/article/10.3390/molecules29215195/s1, Figure S1: Raman spectra for NiFeS@MoS2@rGO; Figure S2: XPS full survey spectrum of NiFeS@MoS2@rGO; Figure S3: BET measurement; Figure S4: Comparison of the EIS for all samples; Figure S5: Representation of two-electrode configuration and CV measurement; Figure S6: specific capacity calculations; Figure S7: Stability of the electrode.
