# Supplementary material for: Design and Optimization of MoS2@rGO@NiFeS Nanocomposites for Hybrid Supercapattery Performance and Sensitive Electrochemical Detection
Source: Molecules. 2024 Nov 2;29(21):5195. doi: 10.3390/molecules29215195 (PMC11547647; doi:10.3390/molecules29215195)
Supplement: Supplementary file 1 [file molecules-29-05195-s001.zip › molecules-3266655-supplementary.pdf]

Design and Optimization of MoS_2@rGO@NiFeS Nanocomposites for Hybrid Supercapattery Performance and Sensitive Electrochemical Detection

Aneeqa Yasmeen[1], Amir Muhammad Afzal*[1], Areej S. Alqarni[2], Muhammad Waqas Iqbal[1], Sohail Mumtaz[3]

[1]Department of Physics, Riphah International University, Campus Lahore, Pakistan

[2]Department of Physics, College of Science, Princess Nourah Bint Abdulrahman University, P.O. Box 84428, Riyadh 11671, Saudi Arabia

[3]Department of Electrical and Biological Physics, Kwangwoon University, Seoul, South Korea

*Corresponding Author: amir.afzal@riphah.edu.pk

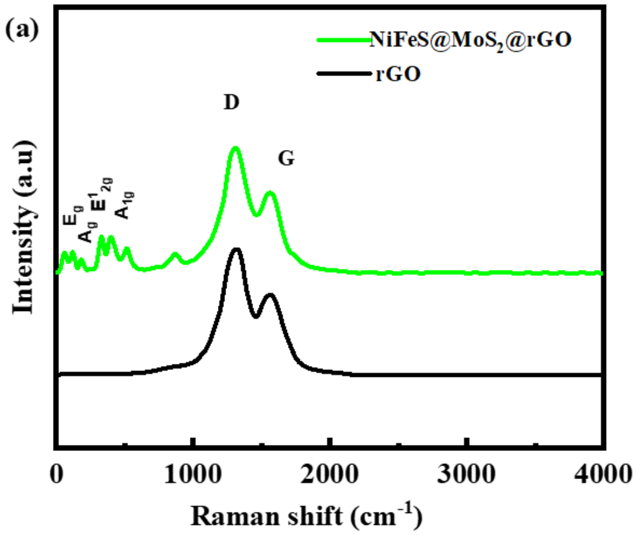

Figure S1: Raman spectra for NiFeS@MoS_2@rGO **.**

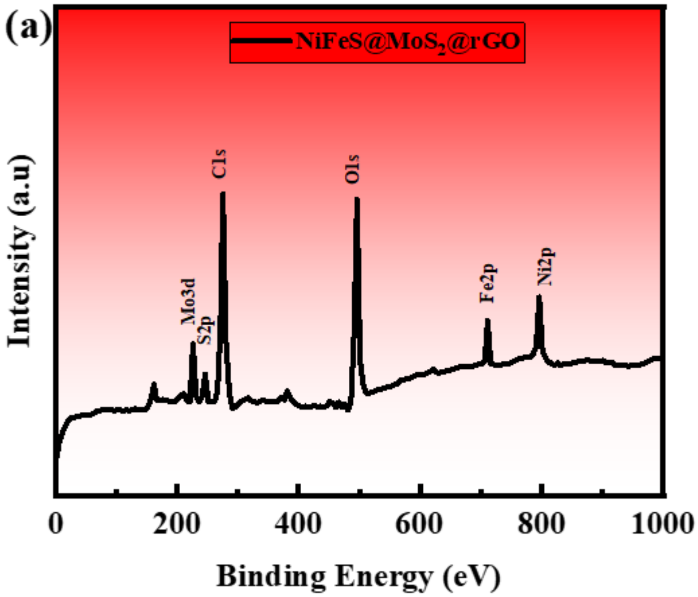

Figure S2: XPS full survey spectrum of NiFeS@MoS_2@rGO.

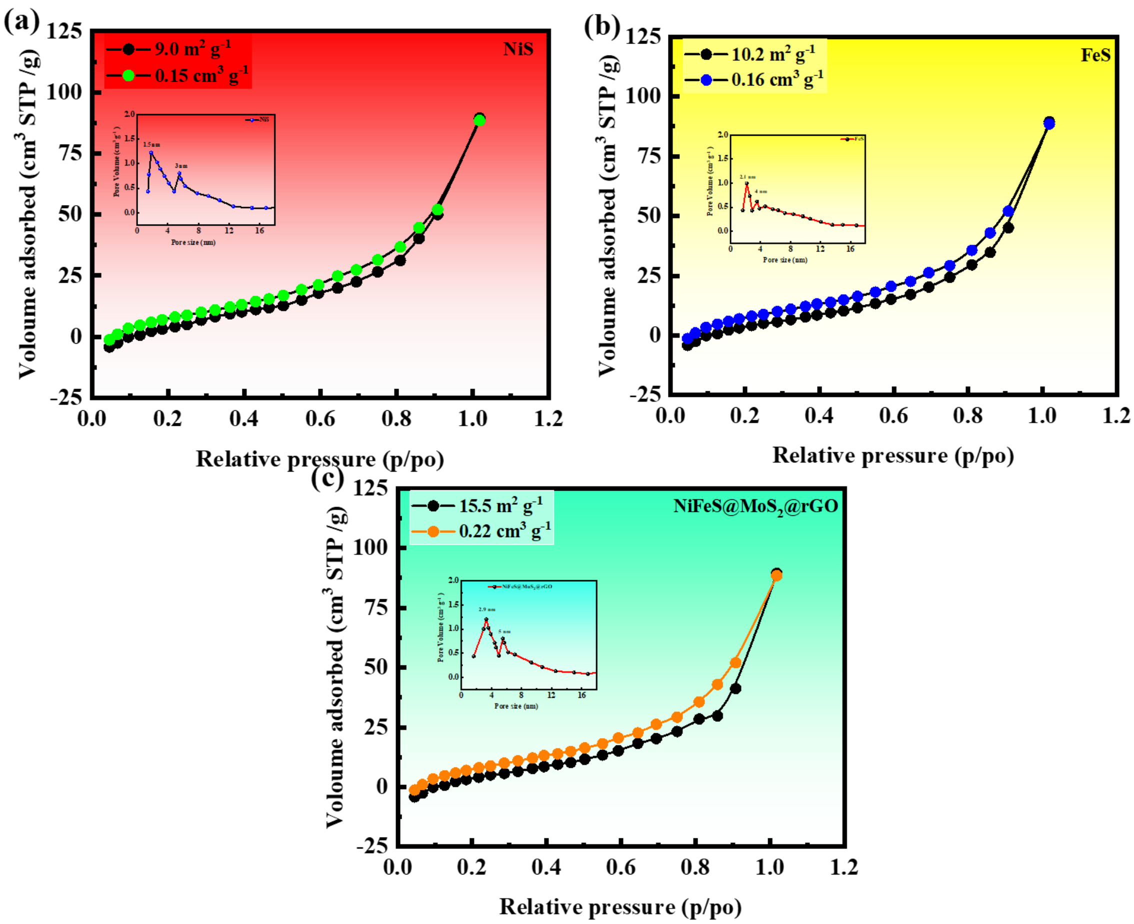

Figure S3:BET measurement (a)NiS.(b)FeS.(c)NiFeS@MOS$_2$@rGO **.**

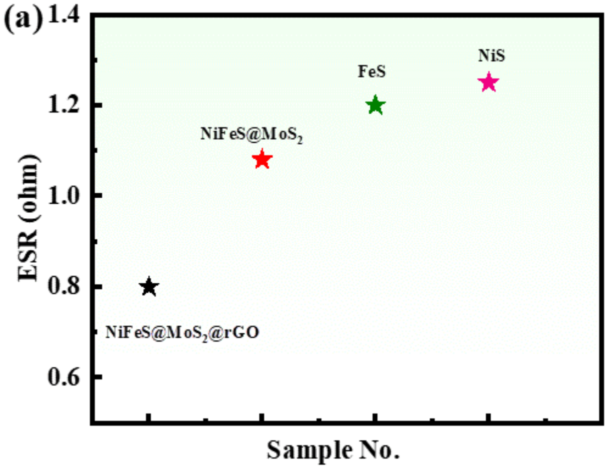

Figure S4: Comarision of the EIS for all samples **.**

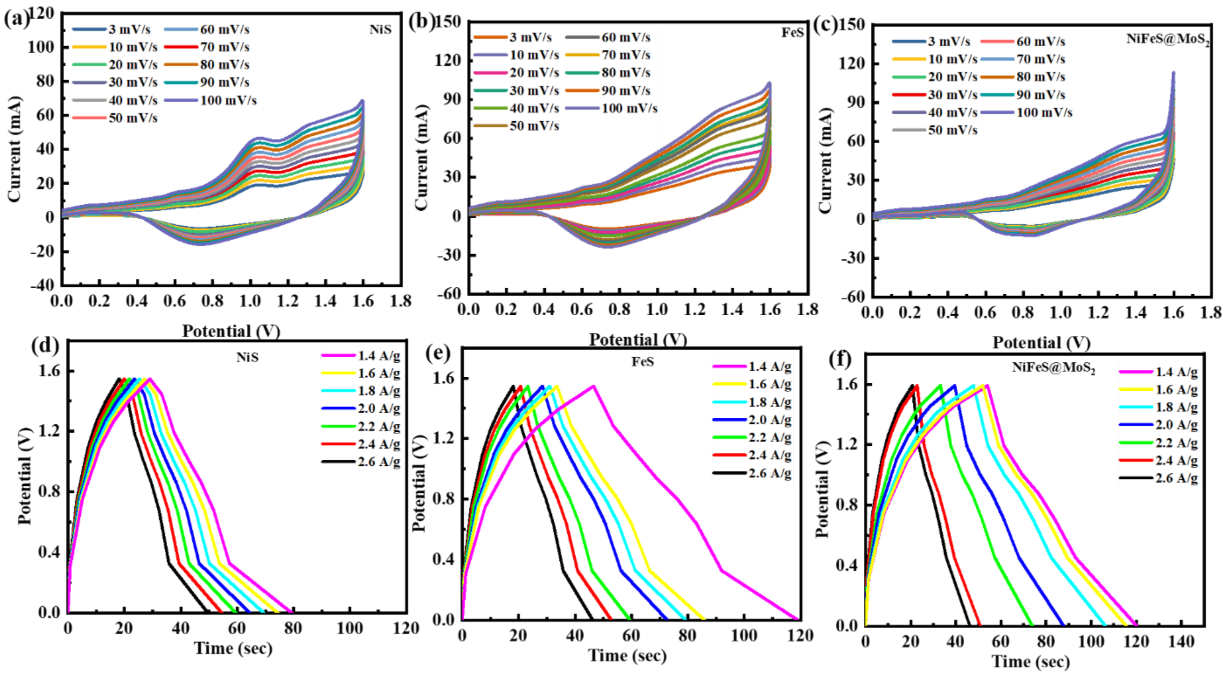

Figure S5: Representation of two-electrode configuration (a) (NiS//AC) (b) CVcurves for (FeS//AC) electrode.(c)CV measurmetns for (NiFeS@MoS_2//AC)(d)GCD analysics for (NiS//AC)(e) Measurment of GCD for (FeS//AC) (f) GCD measurmetns for (NiFeS@MoS_2//AC)

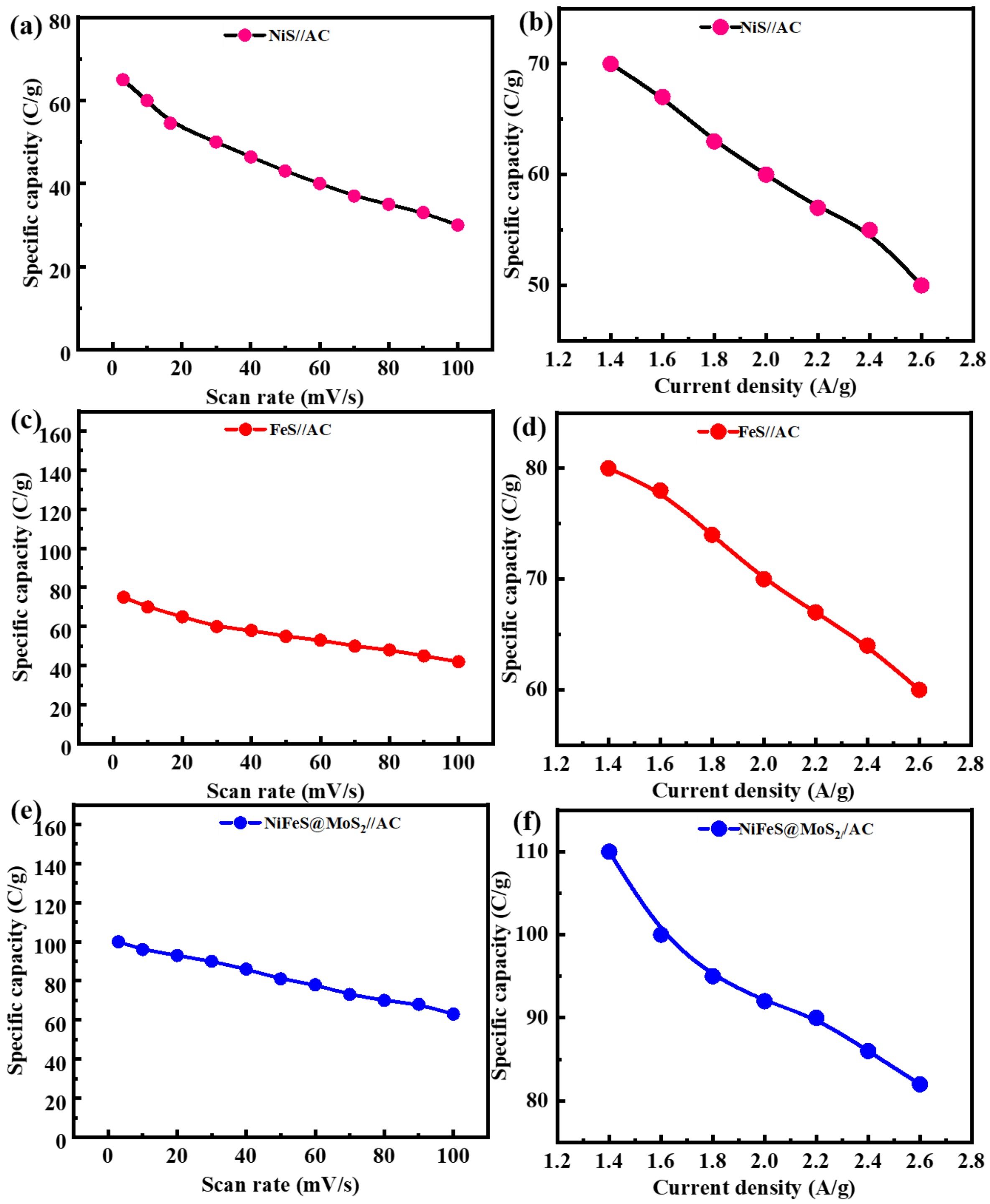

Figure S6: (a)specific capacity of (NiS//AC) by CV analysis.(b) Specific capacity measurement for ((NiS//AC) by GCD analysics. (c)Specific capacity of (FiS//AC) by CV analysics.(d) Specific

capacity measurement for ((FiS//AC) by GCD analysics.(e)Specific capacity of ($NiFeS@MoS_2$//AC). by CV analysis. (f)Specific capacity of ($NiFeS@MoS_2$//AC). by GCD analysis.

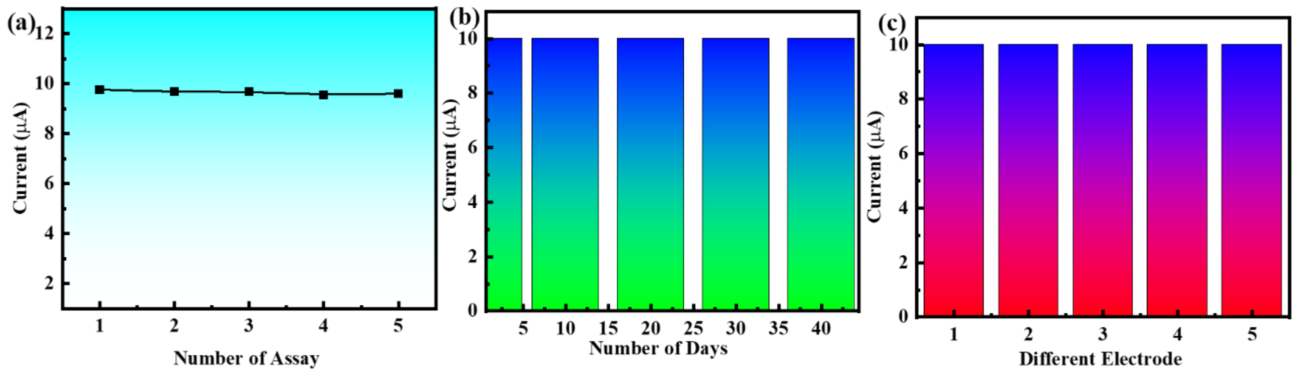

Figure S7: (a) Repeatability; (b) Stability; and (c) Reproducibility for DA sensing.
